# How to Amend the Insurance Bill

**Published:** 1911-12-16

**Authors:** 


### HOW TO AMEND THE INSURANCE BILL.

This Bill, as submitted to the House of Lords, had several changes in the numbering of its clauses. We therefore find it necessary, to avoid confusion, to republish from pages 253 and 254 of our last issue the suggested amendments to the Bill which now affect the various clauses as follows:—

**The suggested amendments** to the Bill are as follows:—

Clause 8, section (1) (*b*): treatment in hospitals in addition to sanatoria, accidents in addition to disease, and institutional benefit are included.

Clause 10, section (1), line 5: institutional benefit is substituted for sanatorium benefit, and also in line 9.

Clause 12, section (2) (*b*), line 2: provision is made for the addition of the word "hospital" after the word "sanatorium"; and in the same clause, section (*c*), line 2, after the word "inmate," the following words are added: "otherwise than as a person in receipt of institutional benefit."

Clause 14, section (1), line 5, "institutional" is substituted for "sanatorium" benefits.

Clause 16, section (1), line 1, "institutional" is substituted for "sanatorium" benefit; and in (1) (*a*), line 2, after the words "such disease" the words "or accident," and line 3, after the word "sanatoria" the word "hospitals" are inserted. In line 5 (1) (*a*), after the word "sanatoria" the word "hospitals" is inserted. In the same clause, section (1) (*b*), line 2, the word "hospitals" is inserted after the word "sanatoria." In the same clause, section (2), line 2, the word "sanatorium" is struck out and the word "institutional" is inserted. In section (3) of the same clause the word "sanatorium" is struck out and the word "institutional" inserted. At the end of this section the following words are added: "Provided that, where a duly qualified member of the medical staff of any such hospital as aforesaid certifies that an insured person is in urgent need of admission to such hospital, such certificate shall have the same validity, so far as regards the admission and subsequent treatment of such person, as a recommendation by the local Health Committee."

In the same clause, section (4), line 2, substitute the word "institutional" for the word "sanatorium," and in line 4 insert the word "hospital" after the words "any sanatorium."

Clause 20, lines 3 and 4, after the words "think fit," delete the following words "to hospitals and other charitable institutions, or."

The suggested new sub-section would now be added to clause 63 and constitute a new sub-section (5).

